# Acupuncture in improving endometrial receptivity: a systematic review and meta-analysis

**DOI:** 10.1186/s12906-019-2472-1

**Published:** 2019-03-13

**Authors:** Yajing Zhong, Fanzhu Zeng, Wanjun Liu, Jing Ma, Yongge Guan, Yang Song

**Affiliations:** 1 School of Nursing, Guangzhou University of Chinese Medicine, No.232 Waihuan East Road, Higher Education Mega Center, Panyu District, Guangzhou, Postal code:510006 Guangdong China; 2 Zhongshan School of Medicine, Sun Yat-sen University, Guangzhou, Guangdong China; 3 The Third Affiliated Hospital of Guangzhou University of Chinese Medicine, Guangzhou, Guangdong China

**Keywords:** Acupuncture, Endometrial receptivity, Systematic review, Meta-analysis, Randomized controlled trial

## Abstract

**Background:**

This systematic review aimed at summarizing and evaluating the evidence of randomized controlled trials (RCTs) using acupuncture to improve endometrial receptivity (ER).

**Methods:**

We searched 12 databases electronically through August 2018 without language restrictions. We included RCTs of women of infertility due to low ER, and excluded infertility caused by other reasons or non-RCTs. Two independent reviewers extracted the characteristics of studies and resolved the differences through consensus. Data were pooled and expressed as standard mean difference (SMD) or mean difference (MD) for continuous outcomes and risk ratio (RR) for dichotomous outcomes, with 95% confidence interval (CI).

**Results:**

We found very low to moderate level of evidence that acupuncture may improve pregnancy rate (RR = 1.23 95%CI[1.13, 1.34] *P* < 0.00001) and embryo transfer rate (RR = 2.04 95%CI[1.13, 3.70] *P* = 0.02), increase trilinear endometrium (RR = 1.47 95%CI [1.27, 1.70] P < 0.00001), thicken endometrium (SMD = 0.41 95% CI [0.11, 0.72] *P* = 0.008), reduce resistive index (RI) (MD = -0.08 95% CI [− 0.15, − 0.02] *P* = 0.01), pulse index (PI) (SMD = -2.39 95% CI [− 3.85, − 0.93] *P* = 0.001) and peak systolic velocity/ end-diastolic blood velocity (S/D) (SMD = -0.60 95% CI [− 0.89, − 0.30] *P* < 0.0001), compared with medication, sham acupuncture or physiotherapy. Acupuncture was statistically significant as a treatment approach.

**Conclusion:**

The efficacy and safety of acupuncture on key outcomes in women with low ER is statistically significant, but the level of most evidence was very low or low. More large-scale, long-term RCTs with rigorous methodologies are needed.

## Background

The incidence of infertility has increased year by year, which is a common concern worldwide [1, 2]. The widespread use of assisted reproductive technique (ART) has helped many women solve infertility problems [3, 4]. In recent years, ART has been continuously developed, greatly improving the rate of fertilization and cleavage [5]. However, there are still many patients have high quality embryos but still cannot be implanted after multiple transplants, ie, repeated implantation failure (RIF) [6]. The spontaneous abortion rate after pregnancy is still over 50% [7]. After decades of development, in vitro fertilization-embryo transfer technology (IVF-ET) has been widely used and gradually recognized and accepted [5, 8]. The success rate is getting higher and higher, up to 30–50% [9]. Advances in embryo culture techniques and improvements in culture systems have greatly improved fertilization rates and cleavage rates, but embryo implantation (ET) rates are still relatively low. Clinically, higher quality embryos can usually be transferred, but only a few embryos can be successfully implanted in the IVF-ET cycle. In 2007, the success rate of IVF-ET was only 20.1%, which brought multiple stresses on physical, psychological and even financial aspects of infertility patients [2, 4, 9, 10]. Low IVF-ET pregnancy rate is a common problem faced by doctors and non-pregnant couples around the world. Fifty to 75 % of pregnancy losses are due to planting failure [11]. Studies have confirmed that 2/3 of IVF-ET implantation failures are due to low endometrial receptivity (ER) [12, 13]. Therefore, improving ER is the key to infertility and increasing the IVF-ET pregnancy rate [14].

ER refers to the ability of endometrium to accept embryos, ER changes with the menstrual cycle. The normal endometrium only contains ET to the maximum extent within a short and critical period, the “implantation window”, generally 6~10 days after ovulation, which is the 20th to 24th day of the normal menstrual cycle [15]. ER is closely related to infertility, and a good ER is a prerequisite for successful implantation of blastocyst [16], about two-thirds of IVF-ET implantation failures are caused by poor ER that good ER improves the success rate of ET [14, 17]. Thin endometrium is one of the most important factors for low ER, which is of great significance for ET, it has no clear diagnostic criteria yet. It is pointed out that the definition of thin endometrium should be that the endometrial thickness is difficult to successfully support the ET [18]. Thin endometrium refers to the thickness of the endometrium in the middle luteal phase (after ovulation) of 6–10 days is < 7.0 mm [14, 19]. Studies have shown that low pregnancy rate is closely related to the thin endometrium. If the average size of the follicles reaches 18 mm and the endometrial thickness is < 7.0 mm, embryo implantation will be greatly affected. At present, the commonly used Western medicine intervention for ER are estradiol valerate, growth hormone, sildenafil citrate and acetylsalicylic acid, etc., so as to improve ER by improving the intimal thickness, endometrial microcirculation, and increasing the sensitivity of estrogen, to achieve certain clinical efficacy. A large number of clinical trials and animal experiments have confirmed that the adverse state of ER can be improved by improving endometrial morphology, regulating estrogen and progesterone levels, and regulating the expression of factors and genes related to ER [20]. Ultrasound technology has been widely used in the evaluation of ER in ART. Ultrasound indicators for evaluating ER include anatomical parameters (endometrial thickness and endometrial type) and physiological parameters (blood flow in the uterine artery and endometrium) [21]. Endometrial thickness, endometrial pattern and endometrial blood supply are closely related to embryo implantation [21]. Endometrial morphology is always divided into three types: type A, type B and type C. Thin endometrium of type B and type C endometrium are not conducive to embryo implantation and development, while clinical studies have found that endometrium with a thickness of more than 8 mm (type A) is more suitable for embryo implantation and development [22, 23]. The blood supply to the endometrium includes the uterine artery, endometrium, and endometrial blood flow [24]. Studies have indicated that the helical arterial blood flow index is a great indicator for predicting ER. Reducing the bilateral uterine artery and endometrial blood flow impedance can significantly improve the blood flow parameters of the uterine artery [25, 26], increase the uterine blood flow, increase patients’ endometrial thickness, which has positive significance for improving ER, and has a positive effect on embryo implantation rate and clinical pregnancy rate [27, 28]. Endometrium is a multicellular tissue that is affected by ovarian-derived steroid hormones and is a major target organ of estrogen and progesterone. During the implantation window, the estrogen and progesterone secreted by the ovary promote the proliferation and differentiation of endometrial cells, and secrete molecules that affect the development of trophoblast cells [29]. Under the common normal action of estrogen and progesterone, the “acceptance state” of endometrial secretion can be completed, and the blood E_2_ level can be increased, which can improve the ER [30]. However, due to different pharmacological effects, the scope of application is different, and it is necessary to prevent abuse during application, and to pay attention to adverse reactions such as gastrointestinal reactions, cardiovascular accidents and metabolic diseases [31, 32]. Therefore, we need to seek other safer and more effective therapies.

As an effective non-drug therapy, acupuncture has been chosen by many infertile couples as a treatment [33–37].It is reported that “fertility problems” is the second most common health condition for which people choose acupuncture treatment in the UK [38]. Acupuncture treatment of female infertility has been widely used and has been shown to affect the menstrual cycle and up-regulate uterine electromyography, which in turn affects reproductive function [39–41]. Acupuncture has a certain improvement effect on ER, it can improve the endometrial morphology, promote the microcirculation of the film inside the uterus, two-way regulating female progesterone and its receptor, regulate molecular biological regulatory factors related indicators such as integrin αvβ3, LIF, VEGF and HOXA10, which provide good conditions for ET, to some extent, and can increase pregnancy rate [25, 42–47]. In 2002, the British expert Paulus et al. proposed separate acupuncture treatment before and after transplantation, which could improve the uterine blood perfusion, thereby increasing the clinical pregnancy rate [48]. In 2006, Johnson verified the validity of the acupuncture observed by Paulus [49]. Chinese experts reported that the clinical pregnancy rate and live birth rate were significantly improved both 24 h before transplantation and 30 min after ET [50]. Dieterle et al. found that acupuncture in the luteal phase can more than double continuous pregnancy rate [51]. Westergaard et al. showed that acupuncture increased clinical pregnancy and continuous pregnancy rates by about 50% on the day of ET [52]. Although the mechanism of acupuncture for ER is still not clear, but acupuncture has been widely used clinically by practitioners of traditional Chinese medicine to treat ER in China and the efficacy is satisfactory [53, 54]. Acupuncture is a safe, mild and non-invasive treatment with relatively less side effects. The efficacy is better than that of medication, the long-term curative effect needs to be further studied and evaluated.

Many clinical trials of the efficacy of different kinds of acupuncture for ER exist, but no relevant systematic reviews and meta-analyses are on the use. The effectiveness and safety of this treatment remain unclear. We aim to assess the effectiveness and safety of acupuncture for ER, to provide evidence for further enhancing the clinical therapeutic effect on patients with low ER. The study may answer whether acupuncture is exactly safe and effective for patients with low ER.

## Methods

This systematic review and meta-analysis is reported in accordance with the Preferred Reporting Items for Systematic Reviews and Meta-Analyses (PRISMA) Statement and was registered at International Prospective Register of Systematic Reviews (number CRD42018105587) [55].

### Literature search strategy

We systematically searched 12 databases for relevant studies published so far: 6 international, 4 Chinese, 1 Korean and 1 Japanese. We retrieved studies that assessed the safety and effect of acupuncture on ER. Search words were acupuncture (e.g. acupuncture, electroacupuncture) and endometrial receptivity (e.g. endometrial receptivity, uterine receptivity, thin endometrium and thin uterus). We did not apply any date or language restrictions.

We used the following combined text and MESH terms for PUBMED search: ((((((((“Acupuncture”[Mesh]) OR Pharmacopuncture) OR (“Acupuncture Therapy”[Mesh])) OR ((((((((Acupuncture Treatment) OR Therapy, Acupuncture) OR Acupuncture Treatments) OR Treatment, Acupuncture) OR Pharmacoacupuncture Treatment) OR Treatment, Pharmacoacupuncture) OR Pharmacoacupuncture Therapy) OR Therapy, Pharmacoacupuncture)) OR Electroacupuncture)) AND ((((Endometrial receptivity) OR Uterine receptivity) OR Thin endometrium) OR Thin uterus)) AND (((clinical[tiab] AND trial[tiab]) OR “clinical trials as topic”[mesh] OR “clinical trial”[pt] OR random*[tiab] OR “random allocation”[mesh] OR “therapeutic use”[sh])). We searched the databases from the beginning to August, 2018.

### Inclusion and exclusion criteria

*Types of Studies.* All RCTs of different kinds of acupuncture for ER were included, such as traditional acupuncture, warm acupuncture, electroacupuncture and transcutaneous electrical acupoint stimulation (TEAS). Non-randomized trials, quasi-experimental studies, and observational studies were excluded. Animal studies, qualitative studies, letters, news articles, editorials, and commentaries were also excluded.

### Types of participants

#### Inclusion criteria

Clinical trials of participants diagnosed with low ER in infertility. We referred to the diagnostic criteria for infertility formulated by World Health Organization (WHO) in 2002 without any age or race limit: primary infertility or secondary infertility patients who had unprotected sexual life for 1 year but without conception. The menstrual cycle is regular, with normal ovulation during the natural cycle, and when the follicle is mature, the endometrial thickness is < 7.0 mm. The uterus is normal in shape, ART has been or has not been performed. Their husband has normal semen quality and shape. Informed consent has been signed.

#### Exclusion criteria

Studies of patients with severe gynecological diseases (e.g., uterine anatomy abnormalities, uterine malformations, intrauterine adhesions), serious systemic or neurologic disease (e.g., diabetes, AIDS, epilepsy), combination of serious risk such as cardiovascular, liver, kidney and hematopoietic system, or refusal to accept acupuncture treatment were excluded, because of the usual or otherwise complicated history that could affect pregnancy. Patients who were treated with herbal medicine were also excluded because herbal medicine is not a conventional therapy.

#### Types of interventions

Studies of acupuncture for ER were included. Use of different kinds of acupuncture alone as an intervention or with other treatment were included; however, acupuncture which are not based on oriental medicine and meridian theory and moxibustion were excluded.

#### Types of control groups

Conventional therapy generally used for low ER such as routine treatment, medication, sham acupuncture or no treatment were included.

#### Types of outcome measures

In this study we analyzed pregnancy rate, embryo transfer rate, live birth rate, high-quality embryo rate, endometrial thickness, endometrial pattern, serum estradiol (E_2_), helical arterial blood flow index including resistive index (RI), pulse index (PI), peak systolic velocity/ end-diastolic blood velocity (S/D) to evaluate the efficacy of acupuncture.

### Data extraction

Two reviewers (YZ and FZ) separately extracted data, including quality assessment from the retrieved studies. The titles and abstracts were reviewed and articles that did not fit the eligibility criteria were excluded. If the title or abstract appeared to meet the eligibility criteria, the full texts of the articles were obtained for further evaluation. Discrepancies were resolved in a consensus meeting or, if agreement could not be reached, they were resolved by referral to a third reviewer (YS). The independent reviewers extracted and tabulated data using a standardized data extraction form, with disagreements finally interpreted by the corresponding author (YS).

We extracted the following data from each selected study: first author, published year, total number of participants, finished number, age, body mass index (BMI), country where the trial was conducted, duration of infertility, healing period, onset to start of treatment, the details of intervention and control group, outcome indicators and reported adverse events. If the data in a study were insufficient or ambiguous, one reviewer (YZ) contacted the corresponding author by e-mail to obtain further information. Two independent reviewers (YZ and FZ) assessed risk for bias according to the PRISMA recommendations [55].

### Assessment for risk of Bias

Two reviewers (YZ and FZ) independently evaluated the risk of bias among the final included studies using the risk of bias assessment tool by the Cochrane Collaboration [56]. The criteria consists of seven items: selection bias (random sequence generation and allocation concealment); performance bias (blinding of participants and personnel); detection bias (blinding of outcome assessment); attrition bias (incomplete outcome data); reporting bias (selective reporting); and other bias. Each study was evaluated as High, Low, or Unclear risk of bias for each item, and the assessment criteria were based on the Cochrane handbook [56]. Any disagreements between the 2 reviewers were resolved by discussion with the corresponding author (YS) until consensus was reached.

### Statistical analysis

We performed statistical analysis using the Review Manager program (Version 5.3 Copenhagen: The Nordic Cochrane Center, The Cochrane Collaboration, 2014). We integrated studies according to the type of intervention, assessed the pregnancy rate, embryo transfer rate, live birth rate, high-quality embryo rate, endometrial thickness, endometrial pattern, E_2_, helical arterial blood flow index including RI, PI and S/D. Dichotomous data were summarized as risk ratio (RR) and continuous data as mean difference (MD) or standard mean difference (SMD). In order to remove the differences in the use of different measurement methods in the study and to eliminate the influence of units, SMD was selected. Heterogeneity between studies was evaluated by using X^2^ (chi-squared) test with *p*-value of *p* < .05 and I^2^ statistic. I^2^ was used to assess heterogeneity between studies, with≥50% was considered to indicate a substantial heterogeneity [56, 57]. A fixed-effects model would be used if there was no significant heterogeneity between studies, otherwise a random-effects model would be employed and subgroup analysis or sensitivity analysis could be performed to explore heterogeneity [56]. 95%CI were calculated, and p < .05 was regarded as statistical significant [56, 57]. If a substantial heterogeneity was detected, we explored sources of heterogeneity through subgroup analyses. Subgroup analyses were attempted in accordance with the difference of interventions. If no factors were found, we did not perform subgroup analysis or data synthesis, but reported a narrative description of the included studies. We conducted a sensitivity analysis using the leave-one-out approach if there was high heterogeneity between studies. Publication bias would be evaluated through a funnel plot analysis if a sufficient number of trials (10 trials) existed.

### Level of evidence

Grading of Recommendations, Assessment, Development and Evaluation (GRADE) was used to evaluate the level of evidence and summarize every outcome [58]. The level of evidence was classified as 4 levels: high, moderate, low, or very low. Assessment of the level of evidence was done on the following domains: risk of bias, inconsistency, indirectness, imprecision and publication bias. We used the GRADE pro software (version 3.6.1 for Windows, Grade Working group) to carry out this work.

## Results

### Search results

**Fig. 1:**
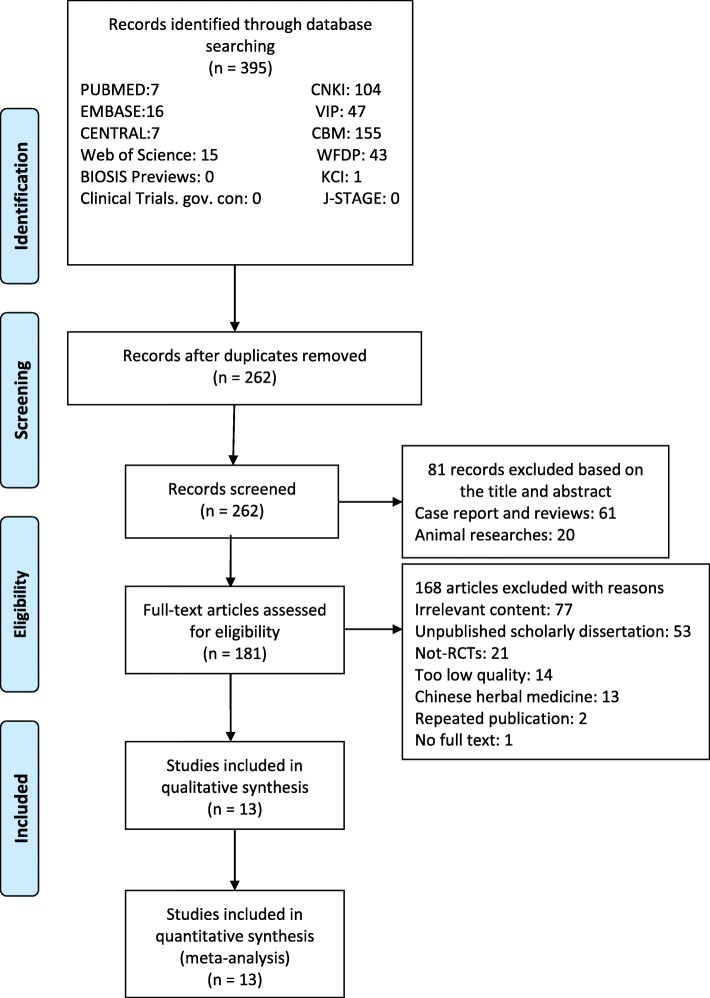
Flow chart

From our database search, we retrieved 395 articles, 7 from PUBMED, 16 from EMBASE, 7 from the Cochrane Central Register of Controlled Trials (CENTRAL), 15 from Web of Science, 1 from Korean Citation Index (KCI), 104 from China National Knowledge Infrastructure Database (CNKI), 47 from VIP Database, 155 from Chinese Biomedical Literature Database (CBM), 43 from WanFang Digital Periodicals Database (WFDP). We did not find related articles in Clinical Trials. gov. con, BIOSIS Previews or Japan Science and Technology Information Aggregator, Electronic (J-STAGE). We screened 262 records after removal of duplicates. Of these, 81 were excluded after reading titles and abstracts: 61 articles were excluded because they were case reports or reviews; 20 were animal experimental research were eliminated. Full texts of 181 articles were downloaded and assessed. During further evaluation, 13 articles were excluded because the use of Chinese herbal medicine; 14 were excluded for too low article quality (published in non-core journals); 53 were excluded for the reason that they were unpublished scholarly dissertations; 77 irrelevant researches and 21 non-RCTs were excluded as well. Two articles were published more than once, 1 full text could not be found. Finally, 13 RCTs were included. The flow chart of the analysis is presented in Fig. 1.

### Included studies and characteristics

The Table 1 showed the main study characteristics. 3041 patients were included in the analysis, of whom 1508 (49.59%) were randomly assigned to the experimental group, and 1533 (50.41%) to the control group. Although we did not impose restrictions on the nation, all these 13 studies were conducted in China and published between 2012 and 2018. Age was ranged from 20 to 45; of these 13 studies, only 5 studies described BMI [59–63]. One study started treatment before menstruation [64]; 3 studies started after menstruation [60, 65, 66]; 4 studies started during the period [61, 67–69]; 1 study started on the fifth day of follicular maturation [70]; and 1study started on the seventh day during the use of gonadotropin (Gn) [63]; 2 studies started before the transplant [62, 71]; and another article did not show the start of treatment [59]. Seven studies were treated for 3 months [59, 61, 64, 65, 67–69], while other studies did not display treatment time, with most studies showing 30 min of treatment at a time. All of these studies used acupuncture with or without other treatment as intervening measure; 7 compared acupuncture with medication [59, 63, 65–68, 70]; 2 compared acupuncture with sham acupuncture [61, 69]; 4 compared acupuncture with usual care and physiotherapy [60, 62, 64, 71]. Two studies applied moxibustion [59, 69], and a study applied press bean [64]. Baseline characteristics among groups were reported as comparable in each study. All except 2 studies count the pregnancy rate of acupuncture based on reported data [69, 70]; 2 reported embryo transfer rate [61, 65]; 2 reported live birth rate [61, 71]; 6 reported endometrial pattern [59, 64–66, 68, 70]; 9 reported endometrial thickness [59–61, 65–70]; 2 reported E_2_ [60, 68]; 2 reported high quality embryo rate [59, 65]; 5 reported RI and PI [59, 63, 64, 66, 69]; 2 reported S/D [59, 63]. The number and period of treatment sessions varied in each study. A summary of the included studies in more detail is presented in Table 1.

**Table 1:** Characteristics of the included studies

Study	Number of participants (Primary/Secondery)	Finished number	Age (y)	Mean age (y)	experiment	control	Duration	Duration of infertility (y)	BMI	Outcomes (primary/secondary outcome)	Starting time
Xiumi You 2018	E:20 C:20	E:20 C:20		E:29.4 ± 4.59 C:28.1 ± 3.89	Warm acupuncture	Medicine	30 min, qod,3 m			Pregnancy rate, Endometrial thickness	7th day of menstruation
Xuemei Chen 2012	E:46 C:27	E:46 C:27	20–45		Acupuncture+ medicine	Medicine	30 min/time,qd		2–12		7th day of Gn
Guoqun Luo 2017	E:25 C:31	E:25 C:31	22–40	E:33.8 ± 4.6 C:32.5 ± 4.1	Warm Acupuncture+ Medicine	Medicine	3 m	E:4.5 ± 2.6 C:4.6 ± 2.3		Pregnancy rate, Endometrial thickness, Endometrial pattern, Embryo transfer rate	After menstruation
Yanhong Li 2018	E:46 C:46	E:46 C:46	35–40	E:37.3 ± 1.2 C:37.5 ± 1.1	Warm Acupuncture+ Acupoint injection+ Medicine	Medicine	qd			Endometrial thickness, Endometrial pattern	5th day of follicular maturation
Bin Zhou 2012	E:80 C:70	E:80 C:70		29 ± 4	TEAS+ Medicine	Medicine	30 min/time,qd	4 ± 3		Pregnancy rate, Endometrial thickness, Endometrial pattern, RI, PI	After menstruation
LiqingYu2018	E:40 C:40	E:38 C:37	21–38	E:30 ± 4 C:29 ± 5	Electroacupunctur+Acupuncture+ Medicine	Medicine	30 min, qod,3 m			Pregnancy rate, Endometrial thickness, Endometrial pattern, E2, PI	Menstruation
Qian Chen A 2015	E:57 C:57	E:57 C:57	24–35	31 ± 3	Acupuncture+ Moxibustion	Medicine	30 min/time,qd,3 m	E:3.61 ± 1.98 C:3.71 ± 2.21	E:22.63 ± 3.23 C:22.79 ± 3.73	Pregnancy rate, Endometrial thickness, Endometrial pattern, RI, PI, High-quality embryo rate	
Qian Chen B 2015	E:20 C:20	E:20 C:20	25–40	E:35.9 ± 2.9 C:35.9 ± 3.4	Acupuncture+ Moxibustion	Sham Acupuncture+ Moxibustion	30 min/time,3 m	E:7 ± 4.4 C:5.3 ± 4		Endometrial thickness, RI, PI	7th day of menstruation
Linxin Zhang 2018	E:14(P:4;S:10) C:12(P:3;S:9)	E:14 C:12	20–45	E:30.3 ± 3.8 C:32.5 ± 4.9	Acupuncture	Routine treatment	30 min, qod	E:5.8 ± 3.9 C:4.5 ± 4.1	E:22.22 ± 3.04 C:20.42 ± 2.11	Pregnancy rate, Endometrial thickness, E2	After menstruation
XiumeiWang2017	E:30(P:17;S:13) C:30(P:18;S:12)	E:30 C:30	E:25–40 C:27–42	E:35.0 ± 3.71 C:34.7 ± 3.18	Warm acupuncture	Press bean+ Electromagnetic wave lamp	3 m	E:6.9 ± 3.02 C:6.5 ± 3.09		Pregnancy rate, Endometrial pattern, RI, PI	10 days before menstruation
Zhenhong Shuai 2015	E:34(P:30;S:4) C:34(P:31;S:3)	E:34 C:34	25–40	E:29.4 ± 3.24 C:29.6 ± 2.60	TEAS	Mock TEAS	30 min/time, 18 times, 3 m	E:4.56 ± 3.25 C:3.88 ± 2.29	E:21.99 ± 2.71 C:22.32 ± 1.64	Pregnancy rate, Endometrial thickness, Embryo transfer rate, Live birth rate	3th day of menstruation
Fan Qu 2017	E:361 C:120	E:333 C:109			TEAS	Routine treatment	30 min			Pregnancy rate, High-quality embryo rate, Live birth rate	Before transplant
Jing Zhong 2017	E:735(P:305;S:430) C:1026(P:4265;S:600)	E:735 C:1026	20–45	E:31 ± 4 C:32 ± 4	TEAS	Routine treatment	30 min/time, 2 times	E:4.8 ± 3.6 C:5 ± 3.6	E:21.8 ± 3 C:21.8 ± 4.5	Pregnancy rate	24 h before transplant

### The assessment for risk of Bias (Figs. 2, 3)

#### Random sequence generation

Among the 13 studies, 10 used computer-programmed random sequencing, random number table or random number generator, and were thus evaluated as low risk of bias [59–61, 63–65, 67–69, 71]; others did not mention the method or detail of random sequence generation, were evaluated as an unclear risk of bias.

**Fig. 2:**
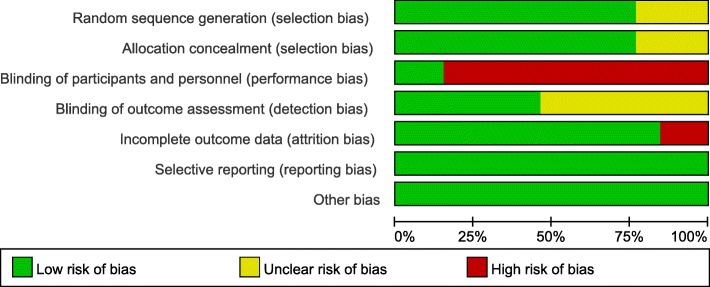
Risk of bias

**Fig. 3:**
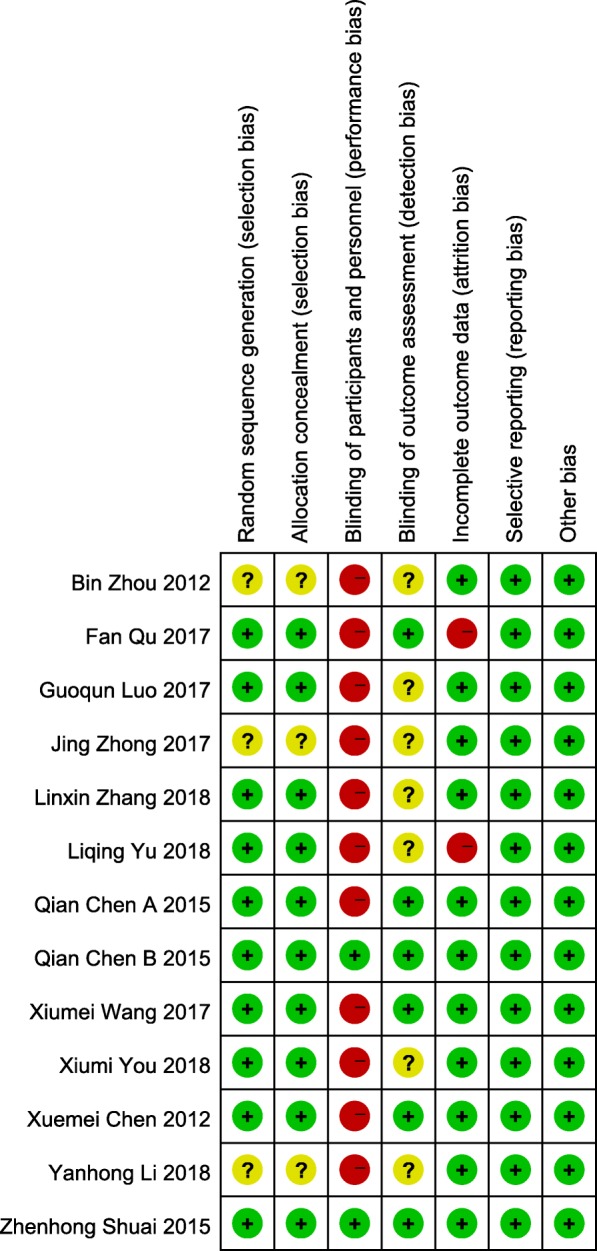
Risk of bias summary

#### Allocation concealment

Of the 13 studies, 10 used sealed-envelops, random list or random assignment method to determine the grouping were given a low risk of bias based on allocation concealment [59–61, 63–65, 67–69, 71]; other 3 studies did not describe the method of allocation concealment were given an unclear risk of bias.

#### Blinding of participants and personnel

Due to the nature of the active control and acupuncture, most of the studies did not perform blinding. Only 2 studies were the participants assessment blinded, using sham acupuncture and mock TEAS as control intervention, resulting in a low risk of bias [61, 69]; the rest were evaluated as high risk of bias that acupuncture was compared to medication, routine treatment or physiotherapy.

#### Blinding of outcome assessment

For outcome blinding, half of the studies adopted single-blind or double-blind methods to assess the intervention measure and consider the blind effect as favorable to have a low risk of bias [59, 61, 63, 64, 69, 71]; other studies were rated as having an unclear risk of bias because insufficient information was provided to determine whether investigators were blinded or not.

#### Incomplete outcome data

Eleven studies have no attrition for missing participants or data were considered as having a low risk of bias [59–67, 69, 70]; the statistical analysis of the 2 studies were not followed by the intention to treat and were rated as a high risk of bias [68, 71].

#### Selective outcome reporting

None of the studies registered protocols, but all studies reported expected outcomes, outcome indicators were complete, and were thus considered all studies as having a low risk of bias.

#### Other sources of Bias

All studies were at low risk of bias for the lack of clear evidence to display other obvious bias.

#### Analysis

RCTs included in this study vary in study designs such as time to start treatment, treatment time, intervention measure and outcome indicators. We categorized these trials according to the types of interventions (acupuncture with or without other treatment versus medication, sham acupuncture or physiotherapy) and outcome indicators.

### Outcomes

We accounted pregnancy rate, endometrial pattern and endometrial thickness as primary outcomes; embryo transfer rate, live birth rate, high-quality embryo rate, E_2,_ RI, PI and S/D as secondary outcomes. We divided the data into two parts according to whether acupuncture was used as a sole treatment or as an auxiliary method.

### Pregnancy rate

**Fig. 4:**
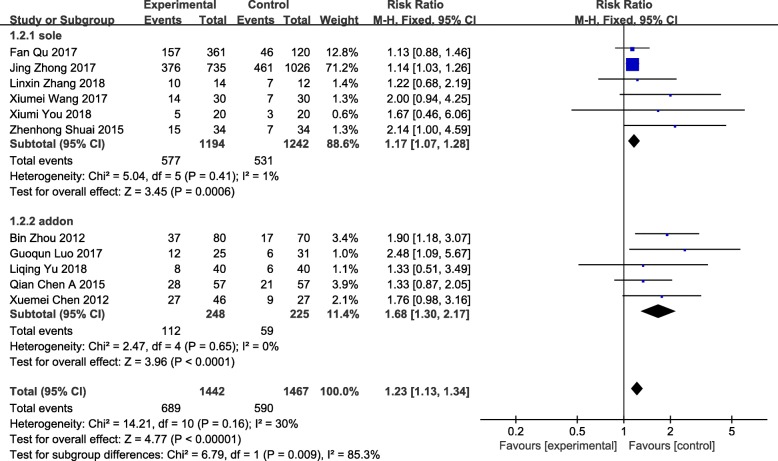
Forest plot of the pregnancy rate

Pregnancy rate was a primary outcome of acupuncture on low endometrial receptivity, which represented the proportion of pregnancies after acupuncture treatment. Obtaining data from 11 studies [59–68, 71], 2909 participants were included. The pregnancy rate had statistical significance (RR = 1.23 95%CI[1.13, 1.34] *P* < 0.00001 I^2^ = 30%) with low heterogeneity in a tolerable level, indicating obvious effect of acupuncture on improving ER while acupuncture versus other treatments (RR = 1.17 95%CI[1.07, 1.28] *P* = 0.0006 I^2^ = 1%) or acupuncture as an auxiliary method versus other treatments (RR = 1.68 95%CI[1.30, 2.17] *P* < 0.0001 I^2^ = 0%) (Fig. 4).

### Endometrial pattern

**Fig. 5:**
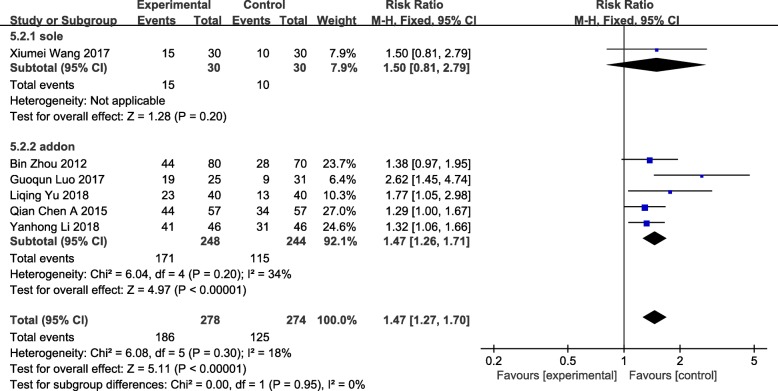
Forest plot of the endometrial pattern

We gathered data from 6 studies including 552 patients, which measured endometrial pattern via color doppler ultrasonography [59, 64–66, 68, 70]. The endometrial morphometry used Gonen classification criteria: Type A: trilinear or multilayered endometrium, strong echo in the outer and middle parts, hypoechoic or dark areas in the inner layer, and obvious linear echo in the uterine cavity; Type B: weak trilinear, isolated echo in the middle, inconspicuous echo in the middle of uterine cavity; Type C: mean strong echo, no intrauterine midline echo [72]. Among them, we affirmed Type B and Type C as non-trilinear endometrium. There was evidence of an increase in trilinear endometrium (Type A) between women who received acupuncture as an addon treatment versus medication or physiotherapy (RR = 1.47 95%CI[1.26, 1.71] *P* < 0.00001 I^2^ = 34%). The difference was statistically significant (RR = 1.47 95%CI[1.27, 1.70] *P* < 0.00001 I^2^ = 18%), low heterogeneity accompanied. The outcome of increase in Type A endometrium was relatively robust (Fig. 5).

### Endometrial thickness

**Fig. 6:**
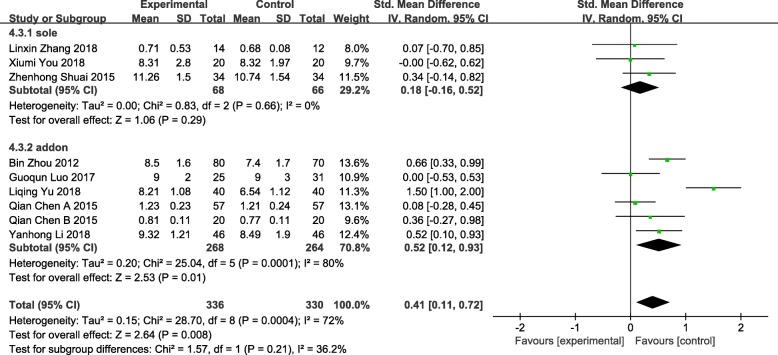
Forest plot of the endometrial thickness

Nine studies reported endometrial thickness involving 666 patients were included, the endometrial thickening was statistically significant (SMD = 0.41 95% CI [0.11, 0.72] *P* = 0.008 I^2^ = 72%) [59–61, 65–70]. The data were analyzed in subgroups according to the difference of interventions. Heterogeneity decreased when the interventions on patients were divided into 2 groups: acupuncture versus medication, sham electroacupuncture or routine treatment, and acupuncture with medication, acupoints injection and/or moxibustion versus medication. Three studies assigned to the group acupuncture versus medication, sham electroacupuncture or routine treatment was not statistically significant (SMD = 0.18 95%CI[− 0.16, 0.52] *P* = 0.29 I^2^ = 0%). Six studies assigned to the group acupuncture with medication, acupoints injection and/or moxibustion versus medication showed a statistical significance (SMD = 0.52 95%CI[0.12, 0.93] *P* = 0.01 I^2^ = 80%), which might be the source of heterogeneity though the heterogeneity was still high, the outcome was relatively steady. Heterogeneity owned, we conducted a sensitivity analysis to look for instability. Significant heterogeneity reduced to I^2^ = 48% when we eliminated the Liqing Yu (2018) study, we reputed that the reason might be intervention measure in this study was conducted during menstruation while others were conducted after or at the end of menstruation. (Fig. 6).

### Embryo transfer rate

**Fig. 7:**
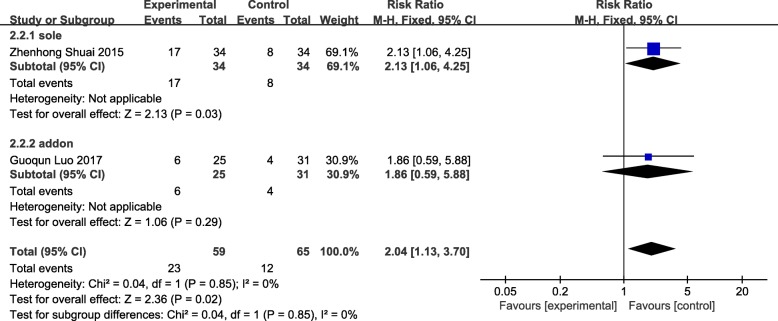
Forest plot of the embryo transfer rate

Two studies were included [61, 65], participants included in both studies had accepted IVF-ET but failed. The comparison of embryo transfer rate among studies were statistically significant (RR = 2.04 95%CI[1.13, 3.70] *P* = 0.02 I^2^ = 0%) without heterogeneity. The results showed that acupuncture was effective in improving the embryo transfer rate, but the reliability of the results was limited due to the small sample size of 124 women as shown in Fig. 7.

### Live birth rate

**Fig. 8:**
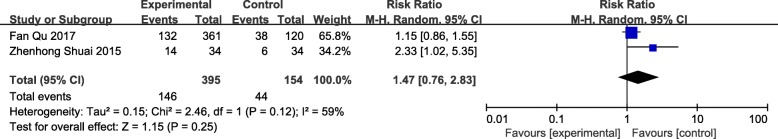
Forest plot of the live birth rate

Two studies measured live birth rate of transplanted embryo were both used acupuncture independently versus sham acupuncture or routine treatment that 549 patients were involved [61, 71], with sufficient subgroup size, a result of RR = 1.47 (95%CI[0.76, 2.83] *P* = 0.25 I^2^ = 59%) was not statistically significant (Fig. 8).

### High-quality embryo rate

**Fig. 9:**
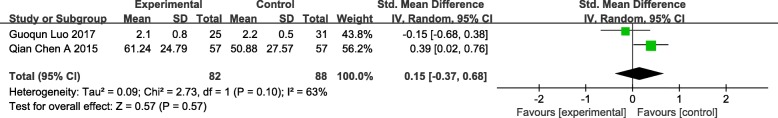
Forest plot of the high-quality embryo rate.

Two studies of high-quality embryo rate including 170 women who have accepted IVF-ET but have failed suggested a not significant difference (SMD = 0.15 95%CI[− 0.37, 0.68] *P* = 0.57 I^2^ = 63%) [59, 65]. We could not find evidence that acupuncture had efficacy in increasing high-quality embryos (Fig. 9).

### E_2_

**Fig. 10:**
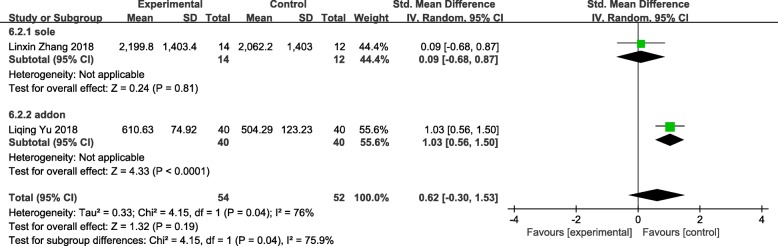
forest plot of the e_2_

Two studies reported E_2_ were brought in [60, 68], 106 patients included. Studies assigned to the group showed a result of not statistically significant (SMD = 0.62 95%CI[− 0.30, 1.53] *P* = 0.19 I^2^ = 76%) that acupuncture should not improve level of E_2_ (Fig. 10)_._

### Helical arterial blood flow index

We displayed 3 indicators of the helical arterial blood flow index to measure changes in the uterus after acupuncture treatment, including RI, PI and S/D.

### RI

**Fig. 11:**
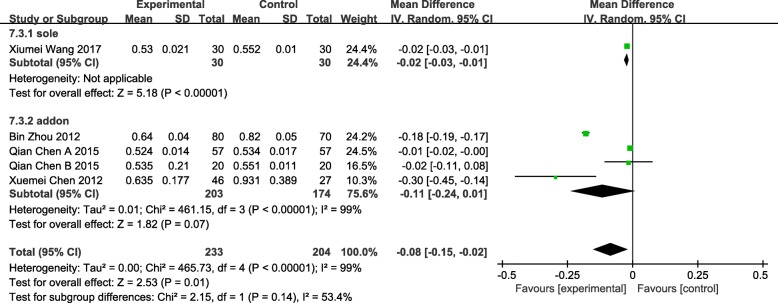
Forest plot of the RI

Five studies of 437 patients described the reduction of RI showed a statistical significance (MD = -0.08 95%CI[− 0.15, − 0.02] *P* = 0.01 I^2^ = 99%) with considerable heterogeneity [59, 63, 64, 66, 69]. Studies were analyzed in subgroups according to interventions: 1 were assigned to the group “sole” which means acupuncture was used as an independent method compared with press bean plus electromagnetic warm lamp in the experimental group (MD = -0.02 95%CI[− 0.03, − 0.01] *P* < 0.00001), the rest were assigned to the group “addon” which means acupuncture was used as an adjuvant therapy with medication or moxibustion versus medication or sham acupuncture plus moxibustion (MD = -0.11 95%CI[− 0.24, 0.01] *P* = 0.07 I^2^ = 99%) that showed not statistical significance. (Fig. 11).

### Pi

**Fig. 12:**
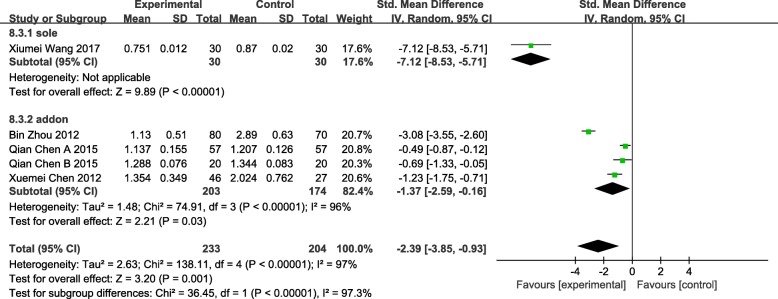
Forest plot of the PI

We gathered data from 5 studies which measured PI [59, 63, 64, 66, 69], eviden1ce of a reduction in PI was statistically significant (SMD = -2.39 95%CI [− 3.85, − 0.93] *p* = 0.001 I^2^ = 97%). Heterogeneity decreased when the interventions on patients were divided into 2 groups: acupuncture was used as an independent or adjunctive treatment. One study assigned to the group “sole” showed a statistical significance (SMD = -7.12 95%CI [− 8.35, − 5.71] *p* < 0.00001); 4 studies assigned to the group “addon” illustrated a significant difference as well (SMD = -1.37 95%CI [− 2.59, − 0.16] *p* = 0.03 I^2^ = 96%). Sensitivity analysis was conducted, heterogeneity decreased when we eliminated the Bin Zhou (2012) study, though it was still significant as I^2^ = 61%. We considered that maybe the Bin Zhou (2012) study used TEAS while others used traditional acupuncture as intervention measure that caused the difference. (Fig. 12).

### S/d

**Fig. 13:**
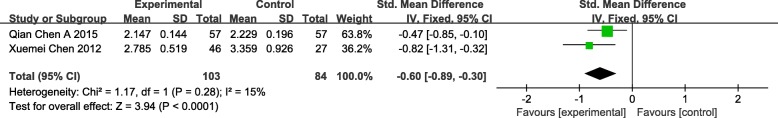
Forest plot of the S/D

Two studies illustrated the effect of acupuncture as an adjunctive therapy involving 170 patients distributed to S/D showed statistical significance (SMD = -0.60 95%CI [− 0.89, − 0.30] *p* < 0.0001 I^2^ = 15%) with sufferable heterogeneity [59, 63], indicating a conspicuous reduction of S/D via acupuncture treatment (Fig. 13).

### Adverse events

There were 3 studies reported adverse events [60, 68, 71]. Only 1 case of fainting during acupuncture treatment was reported in the experimental group [60]; 1 study reported that 3 of the 40 patients (7.5%) in the control group got gastrointestinal indigestion, no adverse events in the experimental group [68]; another study stated neither the experimental group nor the control group owned adverse events [71].

### Publication Bias

**Fig. 14:**
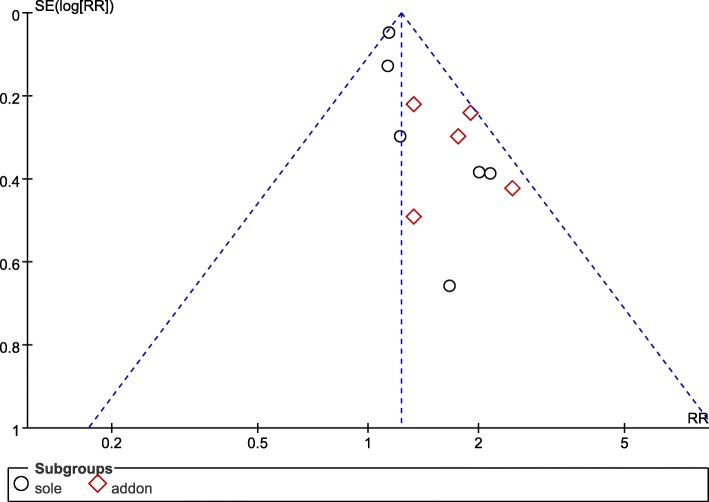
Funnel plot of the pregnancy rate

Publication Bias Evaluation on pregnancy rate of acupuncture was conducted using RevMan (Version 5.3). The improving of pregnancy rate was analyzed through funnel plots, which included 11 trials and 2909 objects. All of the included studies were from China and most of the sample sizes were small, negative results were rarely reported. Results revealed that the distribution of included studies was asymmetric on both sides of the funnel plots, indicating that it may have publication bias in the pregnancy rate of acupuncture (Fig. 14).

### Level of evidence

Overall the quality of evidence accessed via GRADE for comparisons was very low to moderate, most of which was very low to low, limiting our confidence in trial findings.

We rated all studies an evaluation of high risk of bias in at least one domain. We rated a large proportion of studies as having a low risk of bias related to sequence generation and allocation concealment that they conducted “random” properly, except 1 did not mention random method was assessed as having a high risk of bias [62]. High risk of bias was most frequently related to the domains of blinding and attrition which caused reviewers to downgrade evidence a level for the whole comparisons. Reporting bias and other bias were all rated as low risk of bias that required results were covered and no other obvious bias.

Several comparisons showed substantial (I^2^ > 50%) heterogeneity, and the comparison of RI and PI had considerable heterogeneity of I^2^ = 99% and I^2^ = 97%. Although most of the heterogeneity could be explained by different interventions themselves, substantial heterogeneity was often significant enough to result in downgrading of the level of evidence. We were unable to examine the effect of study quality through a sensitivity analysis that we found only 2 studies held a low risk of bias [61, 69]. Some of the comparisons were in a poor quality of consistency, half of which had been downgraded at least a level of evidence due to significant heterogeneity. Most of them caused imprecision and publication on account of small sample sizes and neglected adverse events. Comparisons among studies were conducted directly that indirectness did not downgrade **(**Table 2**)**.

**Table 2:** Level of evidence

Variable	Effect(RR/MD/SMD)	95%CI	*P*	I2(%)	P(X2 test)	Statistical Method	Studies (N)	Sample size (N)	Level of evidence
Pregnancy rate	1.23	1.13, 1.34	< 0.00001	30	0.16	Fixed effects models	11	2909	Very low
Endometrial pattern	1.47	1.27, 1.70	< 0.00001	18	0.30	Fixed effects models	6	552	Moderate
Endometrial thickness	0.41	0.11, 0.72	0.008	72	0.0004	Random effects models	9	666	Low
Electroacupuncture	0.83	0.22, 1.43	0.008	83	0.003	Random effects models	3	298	Low
Acupuncture	0.20	−0.01, 0.40	0.06	0	0.56	Random effects models	6	368	Moderate
Embryo transfer rate	2.04	1.13, 3.70	0.02	0	0.85	Fixed effects models	2	124	Very low
RI	−0.08	− 0.15, − 0.02	0.01	99	< 0.00001	Random effects models	5	437	Very low
With moxibustion	−0.01	− 0.02, − 0.00	0.0006	0	0.90	Random effects models	2	154	Low
Without moxibustion	−0.15	− 0.28, − 0.01	0.03	99	< 0.00001	Random effects models	3	283	Very low
PI	−2.39	−3.85, −0.93	0.001	97	< 0.00001	Random effects models	5	437	Very low
With moxibustion	−0.54	−0.86, − 0.22	0.0010	0	0.60	Random effects models	2	154	Low
Without moxibustion	−3.69	−5.93, −1.45	0.001	97	< 0.00001	Random effects models	3	283	Very low
S/D	−0.60	−0.89, − 0.30	< 0.0001	15	0.28	Fixed effects models	2	187	Low

## Discussion

### Summary of Main findings

The purpose of the review is to summarize and evaluate the efficacy and safety of acupuncture treatment through pregnancy rate, endometrial pattern, endometrial thickness, embryo transfer rate, live birth rate, high-quality embryo rate, E_2,_ RI, PI and S/D in patients with low ER. We included 13 studies, 3041 participants into the meta-analysis, which showed significant heterogeneity of acupuncture for comparison with controls such as medication, sham acupuncture and physiotherapy. Most studies treated patients for 30 min once a day or every other day for three months that most treatments were performed at the end of menstruation or after menstruation.

Acupuncture was used as an auxiliary role, acupuncture with medication, acupoints injection and/or moxibustion versus medication, the endometrium thickening was statistically significant; when acupuncture was used as a sole treatment versus medication, sham acupuncture and routine treatment, the effect in thickening endometrium was not statistically significant. The increased of trilinear endometrium suggested a visible improvement of acupuncture rather than other treatments, accompanying with low heterogeneity and moderate level of evidence. The thickening of endometrium was statistically significant with considerable heterogeneity while the evidence quality was low to moderate. Improvement of embryo transfer rate compared acupuncture versus medication and sham acupuncture illustrated statistical significance with very low level of evidence, while the other 3 indicators of live birth rate, high-quality embryo rate and reduction of E_2_ were statistically non-significant. The decrease of RI used as an independent treatment versus press bean plus electromagnetic warm lamp, PI and S/D used as an adjunct therapy with medication or moxibustion versus medication showed a statistical significance with heterogeneity and very low to low level of evidence. Data collected, the decrease of RI was not statistically significant when it was used as an adjunct therapy of medication and moxibustion versus medication and sham acupuncture plus moxibustion. Above comparisons were rated as very low to moderate level of evidence, most were very low and low. Results described in the previous paragraph were lacking of sufficiently credible evidence to show the effectiveness of acupuncture in improving ER, the effect is weak so far. Additionally, only 3 studies reported adverse events, 1 adverse event related to acupuncture of fainting was described as having reason to believe that acupuncture was mild and safe, 3 cases of 40 patients in the control group were reported of gastrointestinal indigestion after medication treatment rather than electroacupuncture plus acupuncture and medication treatment in the experimental group. Although the experimental group shows a significantly improved effectiveness in comparison to the control group, further research and studies are needed since many included studies are of low methodological quality. All studies were published in China with a risk of bias that prohibited clear conclusions. The sample size in most studies was too small to verify that reports of adverse reactions were affected. Only 3 studies reported untoward effect, so additional large-scale clinical trials were needed before conclusions reached. Adverse events reported from studies were extremely limited, and within the reported content, it can be concluded that the adverse events from acupuncture were not as serious or severe as other control groups. Even though acupuncture is free from the risk of grievous adverse events, most serious adverse events can be prevented through mindful and hygienic administration and education. A newly published, large scale randomized controlled trial stated that, among women undergoing IVF, administration of acupuncture versus sham acupuncture at the time of ovarian stimulation and embryo transfer resulted in no significant difference in live birth rates while adverse events reported by 152 women, all of them were minor and acupuncture specific (discomfort and bruising) and were statistically significantly greater in the acupuncture group for discomfort. These findings do not support the use of acupuncture to improve the rate of live births and clinical pregnancies among women undergoing IVF [73]. This conclusion has implications for the efficacy of acupuncture in improving ER.

The mechanism by which acupuncture improves ER is unclear, but the optimistic effect is being confirmed by many studies. It is difficult to draw a definitive conclusion that acupuncture is more effective than other therapies. Acupuncture has been widely used in China and even around the world, it improves ER for many women mildly and safely, and its mechanism and effect are worthy of our in-depth study.

### Limitation

Some limitations and deficiencies exist in the research. Firstly, the follow-up data of treatment to estimate the long-term efficacy are insufficient and further researches are needed. Diversification of research interventions lead to fewer studies when comparing each intervention, accordingly, the sample size for each comparison is also reduced. Secondly, some studies lack details of random sequence generation, allocation concealment or blinding. Considerable heterogeneities among studies owing to different interventions have been handled with subgroup analyses and sensitivity analyses. Thirdly, the analyses do not take different types of needles and level of acupuncturists into account, which may affect findings and results. Without restrictions of nation or language, all included studies were conducted in China, potential publication bias might exist. In addition, very few negative results and unavailable data may cause bias as well. We did not compare the difference in efficacy between acupuncture and drugs, sham acupuncture or physiotherapy, nor did we compare the effects of acupuncture plus routine treatment with routine treatment simply. Due to the small number of studies we included, it is hard to conclude results accurately.

## Conclusion

Overall, this systematic review and meta-analysis suggests that the effect bases on the use of acupuncture for improving the pregnancy rate and embryo transfer rate, increasing the number of trilinear endometrium, thickening endometrium, and reducing RI, PR and S/D is weak. Acupuncture has relatively fewer side effects on improvement of ER and is comparatively safe. However, the sample size of the very studies is not large enough and the evidence of high quality if insufficient. Therefore, large-scale, long-term RCTs with rigorous methodological quality are needed to clarify the role of acupuncture in ER. Further research is needed to explore long term efficacy and the mechanism of action of the intervention.

## Abbreviations

ART: assisted reproductive technique
BMI: body mass index
CBM: Chinese biomedical literature database
CENTRAL: the cochrane central register of controlled trials
CI: confidence interval
CNKI: China national knowledge infrastructure database
CPR: cardiopulmonary resuscitation
E_2_: serum estradiol
ET: embryo implantation
ET: endometrial receptivity
Gn: gonadotropin
GRADE: Grading of recommendations, assessment, development and evaluation
IVF-ET: in vitro fertilization-embryo transfer technology
J-STAGE: Japan science and technology information aggregator, electronic
KCI: Korean citation index
MD: mean difference
MESH: Medical subject headings
PI: pulse index
PRISMA: Preferred reporting items for systematic reviews and meta-analyses
RCTs: randomized controlled trials
RI: reduce resistive index
RIF: repeated implantation failure
RR: risk ratio
S/D: peak systolic velocity/end-diastolic blood velocity
SMD: standard mean difference
TEAS: transcutaneous electrical acupoint stimulation
WFDP: WanFang digital periodicals database
WHO: World Health Organization
